# A Novel Method for Functional Annotation Prediction Based on Combination of Classification Methods

**DOI:** 10.1155/2014/542824

**Published:** 2014-07-16

**Authors:** Jaehee Jung, Heung Ki Lee, Gangman Yi

**Affiliations:** ^1^Samsung Electronics, Suwon, Republic of Korea; ^2^Department of Computer Science & Engineering, Gangneung-Wonju National University, Gangwon, Republic of Korea

## Abstract

Automated protein function prediction defines the designation of functions of unknown protein functions by using computational methods. This technique is useful to automatically assign gene functional annotations for undefined sequences in next generation genome analysis (NGS). NGS is a popular research method since high-throughput technologies such as DNA sequencing and microarrays have created large sets of genes. These huge sequences have greatly increased the need for analysis. Previous research has been based on the similarities of sequences as this is strongly related to the functional homology. However, this study aimed to designate protein functions by automatically predicting the function of the genome by utilizing InterPro (IPR), which can represent the properties of the protein family and groups of the protein function. Moreover, we used gene ontology (GO), which is the controlled vocabulary used to comprehensively describe the protein function. To define the relationship between IPR and GO terms, three pattern recognition techniques have been employed under different conditions, such as feature selection and weighted value, instead of a binary one.

## 1. Introduction

Conventionally biologists have deduced protein functions through manual experimentation, which is time consuming and involves high expenditure [1]. As increasing wealth of genome data such as DNA sequencing and microarrays, it is clear that manual functional annotation cannot be executed, but the needs for automatic annotation of protein function are increased [1]. In particular, as a boom of sequence analysis research areas such as next generation sequence, functional annotation for unknown sequences, and so forth, became one of the very active research areas. As for new proteins that have not yet been revealed experimentally, the protein functions could be automatically annotated by processing [2] if the model was created using the known protein function. If some features are highly related with some biological functions and specific pattern methods can be used for defining the function, the suggested model can be established to automatically assign the function. Therefore, it would be possible to predict the function in much shorter time than that required by the existing method based on experimentation. One of the popular methods for the automatic assignment is to identify a relationship between features and GO terms [3], where GO provides a controlled vocabulary of terms for annotating proteins. The simplest example is GOA [4], which is manually mapping InterPro terms to GO terms by the InterPro team at EBI. However, this approach is based on the manual mapping; thus recently researchers investigate the relationship using various features such as protein domain, microarray, and protein-protein interaction.

Automated gene annotation research often uses functional databases such as protein functional site, protein family, or gene expression. These databases are usually used for the patterns of base sequence and sequence similarity since sequence similarity is usually related to the functional homology. InterPro (IPR) [5] combined several protein family databases such as Prosite, Prints, Pfam, Prodom, SMART, TIGRFams, and PIR SuperFamily, in order to provide the functional analysis of protein. In addition, the InterPro Consortium provides the InterProScan package [6], which gives the InterPro by simply putting sequences. It would therefore be appropriate to use it as a feature to define the functions of an unknown protein. Moreover, unlike the traditional free text description, controlled vocabularies of various types have been employed. Gene ontology (GO) provides a controlled vocabulary of terms for annotating proteins. Every GO term has a unique numerical identifier that represents the gene function. Each GO term is assigned to one of the three categories of molecular functions, biological processes, or cellular components. These terms are organized into a directed acyclic graph (DAG), which provides a rich framework for describing the function of proteins. Each GO term has a more specific GO term (child) and more than one less-specific term (parent). The database is still under development by the GO Consortium and aims to describe the comprehensive features of the genome.

This paper aims to analyze automatic annotation processing methods by comparing the relationship between IPR and GO-utilizing known data and a range of methods. We started by examining the characteristics of the treated dataset as that was very sparse and skewed. We then described the methods used to reproduce the dataset. The suggested method uses the combinatorial analysis of feature selection and three different pattern recognition approaches so that we would be able to analyze the performance and find the optimized options.

## 2. Related Works

In the case of the functional annotation that identifies the function of genome automatically, various studies related to the database and automatic annotation are in progress in order to define the function of genome of various species from human beings to small microorganisms. The studies are in progress to allow for the automatic prediction of the protein function by an easy access through web or automatic installation, and mostly the database to manage this systematically is also in progress as it has continued to be updated. However, the method of defining the protein function automatically is still at the initial phase; thus, the accuracy is not very high. In the case of using the interpro2go that was manually mapped as in GOA [4], mapping is relied for defining the function; thus, the accuracy is not high. Most studies are conducted by small species to increase the accuracy, and the prediction methods for the protein function based on the calculation that has been researched make a judgment mostly by utilizing the similarities of sequence.

As for the most frequently used tools, they include Gotcha [7], OntoBLAST [8], Blast2GO [9], AutoFACT [10], and so forth; Gotcha [7] can have the similarity of sequence and the directed acyclic graph (DAG) of gene ontology; in other words, the parent node can have several offspring nodes. Thus, it is the method of utilizing the property in which the parent node means the functions of more comprehensive meaning. It is the methods of automatically naming GO by assigning a score to GO owned by the genome that is determined to be similar by judging the similarity of sequence. Blast2GO [9] is the method of annotating new protein functions that cannot be known by gene ontology that is owned by a similar sequence after judging the similarity of sequence by utilizing BLAST [11]. It is the prediction model for the accuracy by assigning weights in accordance with the evidence code that is the annotation code of GO at this point. The evidence code means the code of GO to indicate whether it is automatically named (IEA) and it is determined by the similarity (ISS). OntoBLAST [8] is the method of finding possible protein functions from GO, which are obtained also from BLAST search. AutoFACT [10] proposed a fast annotation method by utilizing BLAST with the relevant database.

## 3. Methods

### 3.1. Features of Data

The data to be used is* Saccharomyces cerevisiae*; it is one of yeast fungi; thus, it belongs to the fungus class and it is the most well-known data by the experiments. Since it forms a relatively small dataset as compared with the other species and it already brings out its related function; thus, it would be an appropriate data for establishing a model for the automatic annotation processing. For the extraction of this data, 4,370 proteins could be obtained as a result of searching and extracting* Saccharomyces cerevisiae* only from SWISS-PROT.

The property to be used as a feature to create a model of data is IPR. IPR has the appropriate features for the reference data that include the protein family binding the protein functions in a similar way and the functions of Prosite, Prints, Pfam, Prodom, SMART, TIGRFams, and PIR SuperFamily that play the central role to refer to the functional domain database. GO is utilized as the reference data for defining the function automatically. GO forms a hierarchical structure and divided into the three big classes—cellular component, molecular function, and biological process.

**Figure 1:**
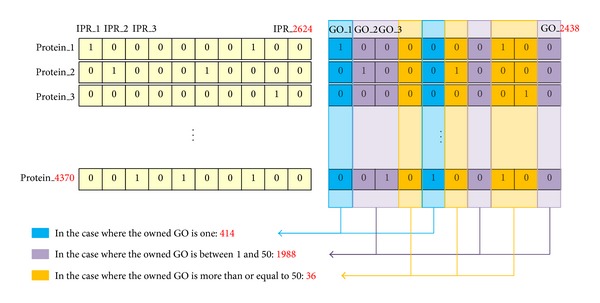
Features of data.

When counting the total number of IPR and GO terms possessed by the 4,370 extracted proteins of* Saccharomyces cerevisiae*, it was found to have 2,624 IPRs and 2,438 GO terms. When this data had one of the properties of IPR or GO term for each protein, it was represented in a binary form. It is represented in a large matrix (4370 ∗ 2438) of GO in a binary form by representing “1” when the proteins have one term of particular GO terms and “0” when they do not have one as parsing gene ontology at ontology in the data section of SWISS-PROT. Also as for IPR, the IPR data was configured in a matrix form of 4,370 ∗ 2,624 by a matrix of binary form as representing whether each protein has it through listing IPRs possessed by* Saccharomyces cerevisiae* proteins after extracting InterPro in the family and domain database section with the same method as described above. A diagram for representing GO of IPR for each protein in a matrix and lining up the quantity of GOs that can be represented by “1” and, in other words, the quantity owned by the proteins would be the same as shown in Figure 1. As shown in Figure 1, it has the problem that it does not have a sufficient quantity for each GO to conduct the learning. When viewed from the perspective of one single GO, the number of cases in which it has only one single GO is 414. This means that only one protein owns the relevant GO; thus, it would not be appropriate to utilize it as the learning data. In addition, the validity was tested through the 10-fold cross validation; thus, GO that has less quantity than a certain level would not be appropriate for the use as the learning data.

However, the biggest problem of the data is that the data exists sparse even though it has a relatively sufficient quantity to be utilized as the learning data. And the fact that the protein data does not have the relevant IPR or GO are inclined to one side than the data having the relevant IPR or GO when viewed from a particular IPR or GO term is also a problem. For instance, only 50 proteins have a particular GO out of 4,370 proteins when viewed from the perspective of a particular single GO; therefore, they are represented by “1” and the remaining 4,320 proteins are represented by “0” since they do not have it. In the case of conducting the learning and experiment with such data, it is quite often predicted that most do not have it since the learning is conducted as being excessively inclined to “0” that is not owned by the learning result; thus, it cannot become an effective model for the automatic function prediction and command processing that has to assign new functions. There are many cases represented by “0” representing “not having” in the case of IPR in addition to GO. This cannot be utilized as an effective feature. Due to such properties of these two features, this paper aims to apply the method as to the feature selection and balanced dataset. Moreover, it aims to analyze the results by converting the binary form of the data into a nonbinary form (weighted IPR) by utilizing the correlation coefficients since the data to be processed is not a binary form.

### 3.2. Prediction Method of Protein Function

This paper aims to compare and analyze the prediction method of protein function by utilizing the data having a sufficient quantity of data as the data of learning and experiment of this paper among the data described in Section 3.1. Before the analysis, it would be essentially required to have a process of reconfiguring it as a balanced dataset due to the feature of not being balanced with the sparseness of data. It aims to compare the case of applying the feature selection by the three mutually different learning methods and the case of conducting the weighted IPR that adds weights to the data, respectively [12–14].

This paper shows comparison and analysis of the prediction methods by the methods presented in Table 1. First as for the learning methods, adaboosting [15] is the method of creating an optimal classification through several times of learning by assigning weights as to the instances wrongly classified by the method of weak learner. SVM is the method of seeking a boundary that makes the error of margin that can differentiate the class to be classified at the hyperplane; thus, it is one of the learning methods of machine learning. SMO [16] is the most well-known tool of libsvm [17]; thus, it can be regarded as the method that has simplified the complexity of SVM by the sequential minimal optimization. As for the methods to be presented in Section 3.2.2, the case of using the feature selection method and the case of not using it were compared and analyzed as W/O in Table 1 meant Without and W meant With. Furthermore, it compared the case of using the method called weighted IPR to be stated in Section 3.2.3. with the case of using the original data as it was.

#### 3.2.1. Dataset Reconfiguration to Adjust Balance

**Figure 2:**
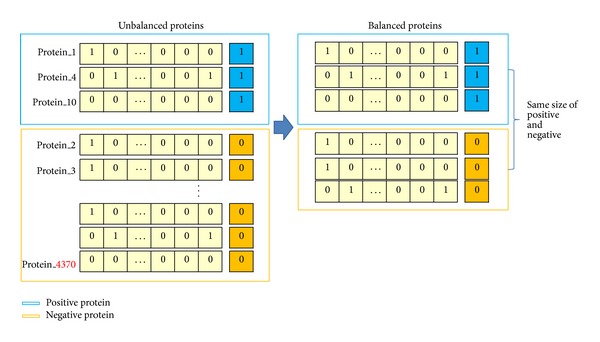
Data reconfiguration using undersampling.

As shown in Figure 2, there are more proteins not having the relevant GO than those having it when viewed based on a particular GO. However, there are more proteins not having a particular GO and, in other words, negative proteins, when learning with such data; therefore, there would be a high degree of probability for the modeling that most of learning results turned out to be not having it. However, it is only possible to find it out by creating a model having the relevant GO rather than a model not having GO. When experimenting with proteins that are not able to perform the function, it is impossible to obtain the desired result. Thus, balanced sampling approach is employed to overcome this handicapped data property [18].

There are the undersampling method and the oversampling method in terms of reconfiguring the data that consists of balanced proteins; the oversampling [19] is the method of making the number equal by generating the data that become the major in terms of quantity as many as the quantities at which the relatively fewer data becomes the major in a random way. The undersampling [20] is the method of meeting the ratio by selecting more data randomly based on the data whose quantity is few. In this experiment, the data that is relatively few in quantity is more important information; therefore, this paper reduces that quantity by utilizing the undersampling. As shown in Figure 2, the data indicated by “1” is to be named as positive protein, whereas the data indicated by “0” is to be named as negative protein. And it is supposed to be trained with proteins that are fewer than 4,370 in terms of the quantity of protein by reconfiguring the data for the learning model at each GO through selecting the negative proteins just as many as the quantities of positive proteins.

#### 3.2.2. Feature Selection

As shown above Figure 1, IPR has the matrix of many binary features of 2,624 when viewed based on one GO. It is the well-known fact that learning and experimenting by selecting only meaningful features would reduce the time to be taken and have a better result as compared with learning and experimenting the method presented above by these many matrices [16, 21–23]. When representing the case in which “1” represents that each IPR has protein by positive data and the case of not having it by “0,” the positive negative data is to be counted for each protein. The positive data is represented as “IPos,” “GPos” and the negative data is represented as “INeg”, “GNeg” at IPR and GO, respectively, and it is possible to classify the state of IPR and GO for each protein. They can become 4 states as shown in Table 2.

**Figure 3:**
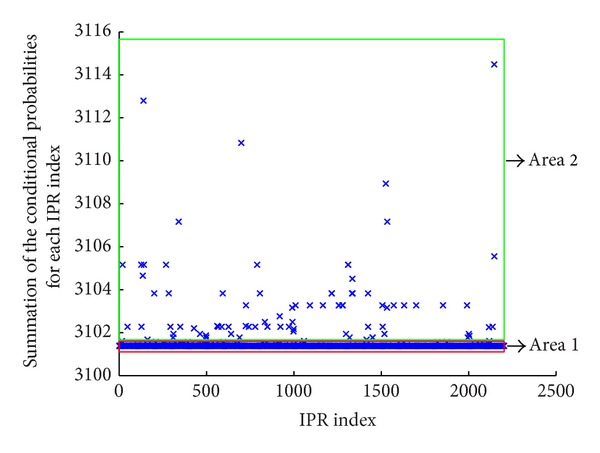
Plot the summation of probability of IPR in terms of GO:0000329.

It is possible to calculate the four probabilities (*N*_GPos_IPos_/*N*_Pos_, *N*_GNeg_IPos_/*N*_Posv_, *N*_GPos_INeg_/*N*_Neg_, and *N*_GNeg_INeg_/*N*_Neg_) by utilizing the 4 data, where *N*_Pos_ stands for the total number of positive proteins and *N*_Neg_ means the total number of negative proteins. These probabilities represent a conditional probability that the gene ontology term may possess depending on the conditions of each IPR. When viewing the property by adding these conditional probabilities as an example of GO:0000329, the diagram as shown below could be viewed. The *x*-axis means several IPRs that are being experimented and the *y*-axis is the value of adding the conditional probabilities. It is possible to see the phenomenon of which most are concentrated in Area 1. On that account, 99 percent of them are those IPRs having negative IPR term and also negative gene ontology (Figure 3). This paper selected the features based on the IPRs that are concentrated in Area 2 as excluding these IPRs. In other words, this is the learning method of utilizing only the selected index as a feature by selecting only the index of IPRs in Area 2 among the 2204 IPRs by calculating the conditional probabilities above for each GO.

#### 3.2.3. Weighted IPR

IPR that is utilized as the feature is the binary data that consists of 0 and 1. When converting this data into a continuous form rather than binary form by utilizing a correlation coefficient, IPR feature data would be expected to select a feature without partiality [24]. This paper aims to analyze the performance between the two methods by the differences between the feature extraction using the binary data that consists of 0 and 1 and the weighted IPR of a continuous form as naming this data as the weighted IPR.

For instance, as shown in Table 3, the table that is composed of 0 and 1 would be modified into a table that utilizes a correlation coefficient (Table 4). A correlation coefficient becomes a value closer to 1 with a higher degree of correlation, whereas it is represented by a value close to 0 when there is no correlation. In addition, it becomes a negative value when there is a mutually contradicting correlation.

This paper aims to change to weight coefficients as proposed by Formula (1) based on this correlation coefficient. First, each protein *P* possesses IPR from 1 to *n*. All the proteins to be experimented are represented by IPR of *n* units. A particular protein having IPR would be represented by 1, whereas those not having IPR would be represented by 0.

For instance, Protein 1 in Table 3 is represented as not having IPR1, IPR5, and IPR6, which are 0, whereas IPR2, IPR3, and IPR4 are represented by IPR possessed by the relevant protein. At this point, there is a relationship between IPR5 and IPR6 since IPR1 is not a property that is not owned when viewed by each IPR of Protein 1. In reference with Table 4, the weight (IPR1) value of IPR1 of corr (IPR1, IPR5) = 0.6124 and corr (IPR1, IPR6) = 0.1667 Protein 1 is 0.6124 + 0.1667 = 0.7791. Moreover, the value of weight sum (IPR1) is represented by IPR1 = 0; therefore, the value of adding all the correlation coefficients of IPR5 and IPR6 becomes 0.7418. Essentially the value was the binary form of 0 and 1 in order to calculate the weighted sum (IPR1) as to IPR1 of Protein 1 of the calculated value; therefore, this finally generates the value of 0.5 ∗ 0.7791/0.7418 = 0.5251 by giving the weighted value 0.5. A new data defined in the new weighted IPR would be generated by such method.  Table 5 can be regarded as one of such cases.

*Converting weighted IPR by the correlations and weights*:

(1) P={IPR1,…,IPRn},Weight(IPRi)=∑j=1|P|corrcoeff(IPRi,IPRj), where  i≠j,Weightsum(IPRi)  =0.5×Weight(IPRi)∑j=1|P|Weight(IPRj),where  IPRi=IPRj.

**Figure 4:**
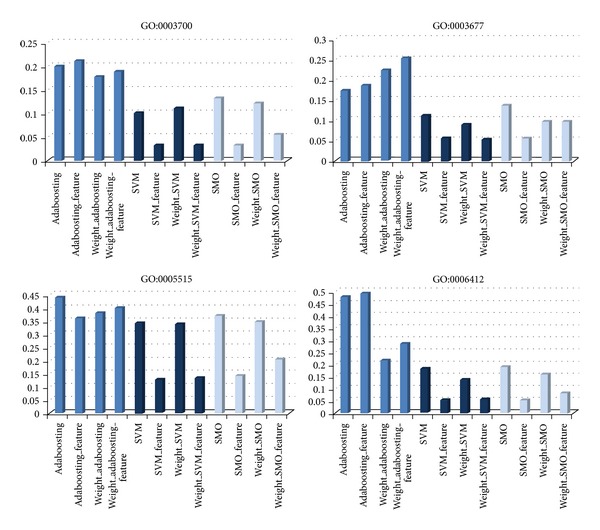
Error rate applying several methods for GO:0003700, GO:0003677, GO:0005515, and GO:0006412.

Figure 4 shows the error rate from applying the 12 methods to GO:0003700, GO:003677, GO:005515, and GO:006412. The GO samples were randomly selected. The three mutually different methods presented in Table 1 are shown in different colors. Better performance is indicated by a smaller error. Blue shows the error rate from adaboosting, where dark blue represents SVM *p* performance and light blue represents SMO. As shown, the error rate for each method varies depending on the GO selection. For example, among the three different methods, SVM has the smallest error rate at GO:0003700 and GO:00367, but SMO has the best performance at GO:006412. Selecting features suggested by SVM or SMO generally resulted in similar or smaller error rates. For the four methods utilizing the original, the feature selection, weighted method, and two combinations, the results for each GO term are as shown in Table 6. This finding shows that they have a high prediction rate ranging from 97 to 99.

**Figure 5:**
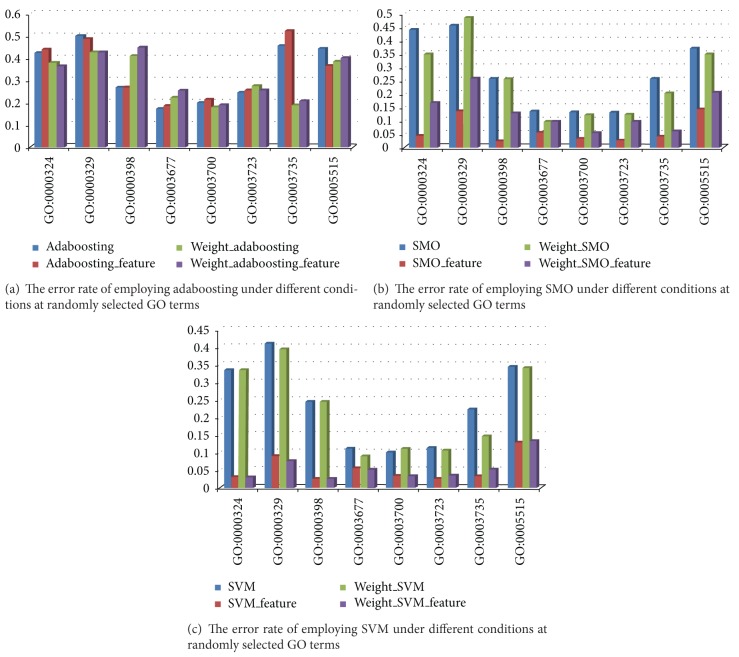
The error rate of three different techniques.

As an extension to Figure 4, Figure 5 shows the error rates dependent on the pattern recognition technique for each GO term. This enables a comparison of the performance under the suggested method, such as feature selection and weighted IPR. The error rate after applying feature selection or weighted IPR is slightly lesser or more than the original dataset, as shown in Figure 5(a). However, for SMO and SVM the errors were dramatically reduced under feature selection (Figures 5(b) and 5(c)).

## 4. Conclusion

This paper compared and evaluated the performance that could define the protein function by applying the classification algorithm by utilizing the feature selection and data transformation. As for the data to be processed, the data having GO term has been composed in much less quantity than the protein not having GO term when viewed by individual GO term. In addition, IPR that is set as the feature point is sparsely distributed; thus, it becomes difficult to learn all the protein data through the general classification algorithm. Due to such limitations, the performance as to the automatic annotation was compared by various classification methods through extracting only the GO term having the standard level or more as the learning subject. Moreover, the performance with the original data was also analyzed by the method of using the binary data as the correlation coefficient through converting it into a newly weighted coefficient.

However, as for the data sampling and feature selection processed in this paper, the GO term is trained primarily the data of protein having a certain amount or more for the learning at* Saccharomyces cerevisiae*, thus, there is the limitation that the quantity of learned data of GO term is small. If it is to be trained by utilizing the data that includes a variety of species such as SWISS PROT in order to overcome this limitation, it will be possible to expect to utilize the automatic function prediction by learning more GO terms with the use of large quantity of data. Thus, this paper aims to study a learning method that is appropriate for this. In addition, it aims to prepare a base to allow for the automatic annotation by seeking for different features that can be utilized as a keyword in addition to IPR when trying to find out unknown protein functions by identifying the correlation with GO.

## Figures and Tables

**Table 1:** Learning methods.

Method	Data			
	Original IPR		Weighted IPR	
Adaboosting	W/O feature selection	W feature selection	W/O feature selection	W feature selection
SVM	W/O feature selection	W feature selection	W/O feature selection	W feature selection
SMO	W/O feature selection	W feature selection	W/O feature selection	W feature selection

**Table 2:** Number of cases in accordance with the status of IPR and GO.

IPR	GO	Express
0	0	*N*_GNeg_INeg_
0	1	*N*_GPos_INeg_
1	0	*N*_GNeg_IPos_
1	1	*N*_GPos_IPos_

**Table 3:** The example of Original IPR.

	IPR1	IPR2	IPR3	IPR4	IPR5	IPR6
Protein 1	0	1	1	1	0	0
Protein 2	1	1	0	0	1	0
Protein 3	0	0	0	0	0	1
Protein 4	1	1	1	0	0	1
Protein 5	0	1	0	1	0	0

**Table 4:** Correlation Coefficient among the IPRs.

	IPR1	IPR2	IPR3	IPR4	IPR5	IPR6
IPR1	1.0000	0.4082	0.1667	−0.6667	0.6124	0.1667
IPR2	0.4082	1.0000	0.4082	0.4082	0.2500	−0.6124
IPR3	0.1667	0.4082	1.0000	0.1667	−0.4082	0.1667
IPR4	−0.6667	0.4082	0.1667	1.0000	−0.4082	−0.6667
IPR5	0.6124	0.2500	−0.4082	−0.4082	1.0000	−0.4082
IPR6	0.1667	−0.6124	0.1667	−0.6667	−0.4082	1.0000

**Table 5:** Weighted IPR.

	IPR1	IPR2	IPR3	IPR4	IPR5	IPR6
Protein 1	0.5251	0.2076	0.1462	0.1462	0.1376	−0.1628
Protein 2	0.2008	0.1295	−0.2501	0.3750	0.1697	0.3750
Protein 3	0.1389	0.3934	0.0889	−0.1334	0.0122	0.5000
Protein 4	0.2633	0.0725	0.2633	0.2500	0.2500	−0.0991
Protein 5	0.7990	0.2500	−0.0633	0.2500	−0.1724	−0.0633

**Table 6:** Error rate for each GO term.

GO	Error rate using SVM with feature selection	Error rate using SMO with feature selection	Error rate using weighted IPR SVM with feature selection	Error rate using weighted IPR SMO with feature selection
GO:0000324	0.030303	0.045455	0.030303	0.166667
GO:0000329	0.090909	0.136364	0.075758	0.257576
GO:0000398	0.025641	0.025641	0.025641	0.128205
GO:0003677	0.055556	0.055556	0.051852	0.096296
GO:0003700	0.033333	0.033333	0.033333	0.055556
GO:0003723	0.026316	0.026316	0.035088	0.096491
GO:0003735	0.033333	0.038889	0.053333	0.06
GO:0005515	0.128571	0.142857	0.144444	0.266667
GO:0005524	0.166667	0.177778	0.144444	0.266667
GO:0005730	0.116667	0.116667	0.108333	0.166667
GO:0005732	0.060606	0.060606	0.060606	0.106061
GO:0005743	0.208333	0.263889	0.208333	0.416667
GO:0005783	0.22069	0.234483	0.224138	0.275862
GO:0005789	0.075758	0.075758	0.106061	0.242424
GO:0005829	0.090909	0.090909	0.1	0.254545
GO:0005886	0.123077	0.123077	0.115385	0.182692
GO:0005935	0.111111	0.111111	0.041667	0.152778
GO:0006281	0.151515	0.181818	0.151515	0.227273
GO:0006355	0.133333	0.15	0.133333	0.166667
GO:0006365	0.075758	0.090909	0.075758	0.166667
GO:0006412	0.05303	0.05303	0.056818	0.079545
GO:0006457	0.030303	0.030303	0.045455	0.166667
GO:0006468	0.009804	0.039216	0.009804	0.058824
GO:0006511	0.030303	0.030303	0.030303	0.121212
GO:0006888	0	0	0	0.083333
GO:0006897	0.013889	0.013889	0.013889	0.152778
GO:0006950	0.090909	0.106061	0.060606	0.212121
GO:0007047	0.083333	0.125	0.092593	0.148148
GO:0009060	0.066667	0.066667	0.066667	0.316667
GO:0009277	0	0	0	0.016667
GO:0016020	0.05	0.05	0.05	0.166667
GO:0016021	0.029412	0.029412	0.019608	0.078431

## References

[1] Jung JH (2008). *Automatic Assignment of Protein Function with Supervised Classifier*.

[2] Elsayed E, Eldahshan K, Tawfeek S (2013). Automatic evaluation technique for certain types of open questions in semantic learning systems. *Human-Centric Computing and Information Sciences*.

[3] Ashburner M, Ball CA, Blake JA (2000). Gene ontology: tool for the unification of biology. The Gene Ontology Consortium. *Nature Genetics*.

[4] Camon E, Magrane M, Barrell D (2004). The gene ontology annotation (GOA) database: sharing knowledge in uniprot with gene oncology. *Nucleic Acids Research*.

[5] Hunter S, Jones P, Mitchell A (2012). InterPro in 2011: new developments in the family and domain prediction database. *Nucleic Acids Research*.

[6] Quevillon E, Silventoinen V, Pillai S (2005). InterProScan: protein domains identifier. *Nucleic Acids Research*.

[7] Martin DMA, Berriman M, Barton GJ (2004). GOtcha: a new method for prediction of protein function assessed by the annotation of seven genomes. *BMC Bioinformatics*.

[8] Zehetner G (2003). OntoBlast function: from sequence similarities directly to potential functional annotations by ontology terms. *Nucleic Acids Research*.

[9] Conesa A, Götz S, García-Gómez JM, Terol J, Talón M, Robles M (2005). Blast2GO: a universal tool for annotation, visualization and analysis in functional genomics research. *Bioinformatics*.

[10] Koski LB, Gray MW, Lang BF, Burger G (2005). AutoFACT: an automatic functional annotation and classification tool. *BMC Bioinformatics*.

[11] Altschul SF, Madden TL, Schäffer AA (1997). Gapped BLAST and PSI-BLAST: a new generation of protein database search programs. *Nucleic Acids Research*.

[12] Malkawi M, Murad O (2013). Artificial neuro fuzzy logic system for detecting human emotions. *Human-Centric Computing and Information Sciences*.

[13] Salim K, Hafida B, Ahmed RS (2014). Probabilistic models for local patterns analysis. *Journal of Information Processing Systems*.

[14] Al-Shahib A, Breitling R, Gilbert D (2005). Feature selection and the class imbalance problem in predicting protein function from sequence. *Applied Bioinformatics*.

[15] Freund Y, Schapire R (1996). A short introduction to boosting. *Journal of Japanese Society for Artificial Intelligence*.

[16] John CP (1998). Sequential minimal optimization: a fast algorithm for training support vector machines.

[17] Chang C, Lin C (2011). LIBSVM: a Library for support vector machines. *ACM Transactions on Intelligent Systems and Technology*.

[18] Jo EB, Lee JH, Park SY, Kim SM (2014). Predicting osteoporosis and osteoporotic fractures by analyzing the fracture patterns and trabecular microarchitectures of the proximal femur. *Journal of Convergence*.

[19] Chawla NV, Bowyer KW, Hall LO, Kegelmeyer WP (2002). SMOTE: synthetic minority over-sampling technique. *Journal of Artificial Intelligence Research*.

[20] Kubat M, Matwin S Addressing the curse of imbalanced training sets: one-sided selection.

[21] Guyon I, Elisseeff A (2003). An introduction to variable and feature selection. *Journal of Machine Learning Research*.

[22] Hong S, Chang J (2013). A new k-NN query processing algorithm based on multicasting-based cell expansion in location-based services. *Journal of Convergence*.

[23] James A, Mathews B, Sugathan S, Raveendran D (2005). Discriminative histogram taxonomy features for snake species identification. *Human-Centric Computing and Information Sciences*.

[24] Kao WH, Liou BS, Shen WH, Tsou YL (2013). Applying Boolean logic algorithm for photomask pattern design. *Journal of Convergence*.

